# Annexin A6 Modulates the Secretion of Pro-Inflammatory Cytokines and Exosomes via Interaction with SNAP23 in Triple-Negative Breast Cancer Cells

**DOI:** 10.3390/cells15111013

**Published:** 2026-05-31

**Authors:** Nobelle I. Sakwe, Olga Y. Korolkova, Ngoc B. Vuong, Alayjha D. Edwards, Perrin J. Black, Destiny D. Ball, Antonisha R. McIntosh, Portia L. Thomas, Diva S. Whalen Melvin, Heather K. Beasley, Antentor O. Hinton, Josiah Ochieng, Amos M. Sakwe

**Affiliations:** 1 Department of Biomedical Sciences, School of Graduate Studies, Meharry Medical College, Nashville, TN 37208, USA; 2 Department of Biology, Tougaloo College, Tougaloo, MS 39174, USA; dwhalen@tougaloo.edu; 3 Department of Molecular Physiology and Biophysics, Vanderbilt University, Nashville, TN 37235, USA

**Keywords:** Annexin A6, pro-inflammatory cytokines, exosomes, extracellular vesicles, SNAP23, cholesterol, secretion, triple-negative breast cancer

## Abstract

**Highlights:**

**What are the main findings?**

- Downregulation of AnxA6 in TNBC cells inhibited while AnxA6 upregulation stimulated the secretion of pro-inflammatory cytokines and cholesterol-enriched exosomes.
- AnxA6 influences the secretion of cytokines and exosomes via interaction with SNAP23, a component of the membrane fusion machinery.
- Blocking extracellular AnxA6 with neutralizing antibodies reduced the viability of AnxA6-low TNBC cells but had little effect on AnxA6-high cells.

**What are the implications of the main findings?**

- Extracellular AnxA6 is critical for the survival of the proliferative AnxA6-low basal-like breast cancer cells.
- AnxA6 promotes TNBC progression by facilitating the secretion of pro-inflammatory cytokines and cholesterol-enriched exosomes.

**Abstract:**

Annexin A6 (AnxA6) is a predominantly intracellular calcium-dependent membrane-binding multifunctional protein that is also detected extracellularly and in small extracellular vesicles (exosomes). We previously demonstrated that lapatinib resistance in triple-negative breast cancer (TNBC) cells is associated with AnxA6 upregulation and accumulation of cholesterol in late endosomes. Here, we investigated the fate of AnxA6 and cholesterol in lapatinib-resistant (LAP-R) cells and whether extracellular AnxA6 influences TNBC cell survival. We demonstrate that reduced expression of AnxA6 in LAP-R cells decreased the secretion of MCP-1/CCL2, CCL8/IL-8, DKK1, TSP-1, and OPN by antibody arrays. The secretion of exosomes was also markedly reduced in AnxA6-depleted LAP-R cells, while AnxA6 upregulation stimulated the release of MCP-1 and exosomes. Compared to the respective controls, exosome-associated AnxA6, Rab7, and cholesterol levels were increased in exosomes isolated from AnxA6-expressing LAP-R cells. Mechanistically, we demonstrated by co-immunoprecipitation, GST pulldown, and proximity ligation assays that AnxA6 interacts with SNAP23, a component of the membrane fusion machinery. Finally, blocking extracellular AnxA6 with neutralizing antibodies reduced the viability of AnxA6-low TNBC cells but had little effect on AnxA6-high cells. These findings suggest that extracellular AnxA6 is critical for the survival of highly proliferative AnxA6-low basal-like breast cancer cells and that AnxA6 influences TNBC progression by facilitating the secretion of pro-inflammatory cytokines and cholesterol-enriched exosomes.

## 1. Introduction

Annexin A6 (AnxA6) is a Ca^2+^-dependent membrane-binding protein that, in triple-negative breast cancer (TNBC), is implicated in tumor progression and tumor metastasis [1,2]. It has also been shown to be involved in the regulation of calcium (Ca^2+^) entry into cells, fatty acid lipid droplet formation, cholesterol homeostasis, and drug resistance [3,4]. Its expression level can tip the balance between lipid storage and lipid mobilization [5,6], metabolic quiescence and metabolic activation [6], as well as between stress survival and susceptibility to metabolic and other therapies [6,7]. In TNBC cells, the expression of AnxA6 is relatively higher in pro-invasive mesenchymal-like cells but it is barely detected or expressed at relatively lower levels in highly proliferative basal-like epithelial breast cancer cells [8]. Previous studies have also shown that the cellular levels of AnxA6 are induced by Ca^2+^ channel blockers [1], tyrosine kinase inhibitors such as lapatinib [9], and physiological cues like hypoxia [7]. Although it lacks a classic signal peptide for secretion, AnxA6 is consistently detected in the extracellular space, mostly associated with extracellular vesicles [10,11], but also as a vesicle-free membrane or extracellular matrix-bound protein [12,13]. Other studies have shown that AnxA6-containing EVs are pro-metastatic and mediate drug resistance by mechanisms that include interactions with cell surface receptors, membrane phospholipids, extracellular matrix (ECM) components, and cytoskeletal proteins such as F-actin. Although these interactions activate signaling pathways including focal adhesion kinase (FAK), nuclear factor kappa B (NF-κB), and autophagy [4,12,14,15,16], the contribution of extracellular AnxA6 to TNBC cell survival remains unclear.

Pro-inflammatory cytokines are known to be secreted into the tumor microenvironment (TME) by several cell types, including infiltrating immune cells, tumor cells, and stroma cells [17]. Some of these factors, such as monocyte chemoattractant protein-1 (MCP-1/CCL2), secreted by tumor and several other cell types, promote the recruitment of monocytes, memory T cells, natural killer (NK) cells, and dendritic cells [18]. Secreted interleukin 8 (IL-8/CXCL8) has also been shown to promote the recruitment of neutrophils to the TME [19,20]. Collectively, these factors promote the development and progression of breast cancers by enhancing inflammation, immune suppression, and angiogenesis [21]. The secretion of cytokines with a signal peptide such as MCP-1/CCL2 mostly occurs via packaging of the *de novo*-synthesized proteins into secretory vesicles or granules, followed by release via receptor-mediated (regulated) or non-regulated (constitutive) exocytosis [22,23]. Cytokines without a signal peptide, e.g., IL-1β, on the other hand, are secreted via non-classical exocytosis, in which they are packaged into multivesicular bodies and released as nano-sized, membrane-bound extracellular vesicles (EVs) [24]. While pro-inflammatory cytokines exert their effects via their specific receptors, EVs, by virtue of their cargo, play diverse but important roles in intercellular and cell–ECM interactions in the TME and beyond [25].

Like other members of the annexin family, AnxA6 translocates to the plasma membrane in a calcium-dependent manner, suggesting that altered expression levels may impact membrane-associated processes such as membrane cholesterol dynamics, vesicle trafficking, and exocytosis [3,26]. Previous reports have also demonstrated that EV secretion can be triggered by cell detachment [27] or plasma membrane damage [28] and that AnxA6 downregulation impairs multivesicular body (MVB) trafficking, causing their accumulation at the cell periphery [28]. Although the potential for annexins, including AnxA6, as mediators of membrane fusion was proposed several decades ago [29], and subsequent evidence suggests that AnxA6 regulates the exocytosis of catecholamines and exosomes [28,30], the underlying mechanisms remain poorly understood.

We recently reported that chronic treatment of AnxA6-low TNBC cells with the dual EGFR/HER2 tyrosine kinase inhibitor lapatinib led to increased expression of AnxA6 and accumulation of cholesterol in the late endosomal compartment. Withdrawal of lapatinib treatment resulted in the return of AnxA6 and cholesterol levels to near-basal levels [9]. In this study, we examined whether the secretion of pro-inflammatory cytokines and EVs in lapatinib-resistant cells is AnxA6-dependent and if extracellular AnxA6 is necessary for the survival of TNBC cells. Our findings suggest that extracellular AnxA6 is critical for TNBC cell survival and that AnxA6 regulates the secretion of pro-inflammatory cytokines and cholesterol-enriched EVs via interaction with SNAP23, a component of the membrane fusion machinery.

## 2. Materials and Methods

### 2.1. Cell Culture

The non-malignant breast epithelial cell line MCF-10A and the TNBC cell lines BT-549, MDA-MB-468, MDA-MB 231, and HCC1806 were all purchased from the American Type Culture Collection (ATCC, Manassas, VA, USA). MCF-10A cells were cultured in human mammary epithelial cell (HuMEC) medium supplemented with HuMEC supplement containing epidermal growth factor, hydrocortisone, isoproterenol, transferrin, and insulin, as well as 25 mg of bovine pituitary extract (Gibco, Life Technologies, Grand Island, NY, USA). BT-549 and HCC1806 were cultured in Dulbecco’s modified Eagle’s nutrient F-12 (DMEM/F12) medium, MDA-MB 231 was grown in RPMI 1640, and MDA-MB-468 in Leibovitz’s L15 media (Gibco, Life Technologies, Grand Island, NY, USA). All media were supplemented with 10% fetal bovine serum, NaHCO_3_ (10 mM), penicillin (100 units/mL), and streptomycin (50 units/mL). The cells were maintained in a humidified 95% air and 5% CO_2_ incubator at 37 °C and media were changed every 2–3 days. Where indicated, serum starvation was carried out by culturing the cells in the respective base medium supplemented with 0.5% FBS and the indicated antibiotics. For treatment of cells with the indicated compounds, the cells were trypsinized, seeded at the desired cell density, and allowed to attach overnight in complete medium. The following day, the media were aspirated and replaced with fresh media containing the indicated concentrations of the drugs and cultured for the indicated times.

### 2.2. Plasmid Transfection

The generation of AnxA6-downregulated MDA-468 and BT-549 cells, as well as AnxA6-overexpressing MDA-468 and HCC1806 cells, was previously described [8,9,10]. Briefly, cells were transfected with non-silencing control (NSC) and shRNAs (A6sh2 and A6sh5) cloned into the pGIPZ lentiviral vector (Horizon Discovery, Waterbeach, UK), targeting the coding sequence of AnxA6. For ectopic expression of AnxA6 in the AnxA6-low MDA-468 cells, the cells were transfected with empty vector (EV) and Flag-tagged AnxA6 (A6) cloned in plasmid pLV[Exp]-EGFP:T2A:Puro-CMV (VectorBuilder Inc., Chicago, IL, USA). Transfected cells were sorted for green fluorescent protein expression and then selected with puromycin for three to five weeks. The stably transfected MDA-468 cells were then treated with lapatinib (2 µM) over several months to generate lapatinib-resistant cells (LAP-R). To generate lapatinib-withdrawn cells (LAP-RW), the cells were cultured without lapatinib for five days as previously described [9], during which AnxA6 cellular levels returned to near-basal levels.

### 2.3. Culture Supernatant

Cells were cultured to 80% confluency passaged, and equal numbers of cells (~1–2 × 10^6^ cells/mL) were seeded in T-75 flasks and incubated overnight at 37 °C. The following day, cells were washed twice with room-temperature PBS and cultured in 1 mL of serum-free medium overnight. The culture supernatant was transferred into new tubes, cleared by centrifugation at 275× *g* for 5 min at 4 °C, and aliquoted into 2 mL tubes and stored at −80 °C or used in antibody arrays. The cells were also harvested by trypsinization and counted using a TC20 automated cell counter (Bio-Rad Laboratories, Hercules, CA, USA).

### 2.4. Cytokine Array

Cytokine profiling was performed by using the Proteome Profiler Human XL Cytokine Array Kit according to the manufacturer’s instructions (Cat #ARY022B, R&D Systems, Inc., Minneapolis, MN, USA), using cleared culture supernatants collected from lapatinib-resistant AnxA6-expressing (NSC-R) control and AnxA6-depleted (A6sh-R) MDA-468 cells. The blots were revealed by enhanced chemiluminescence (ECL) (Perkin Elmer, Waltham, MA, USA), and the intensity of the spots was quantified by using the particle analysis module in the ImageJ v1.54d software, as described recently [31]. Briefly, the densitometric values from the duplicate spots representing each cytokine were normalized to the array controls in each membrane, and the differences in the detected cytokines between the NSC-R control and A6sh-R cells were expressed as fold changes (FCs). Proteins with FC > 1 were considered upregulated, while those with FC < 1 were downregulated.

### 2.5. Isolation of Small Extracellular Vesicles (Exosomes)

Cell culture supernatants harvested as described above were centrifuged at 1500× *g* for 4 min to remove cells and cell debris and filtered through 0.45 µm filters. Exosomes were isolated from the cleared culture supernatants by differential velocity centrifugation, as previously described [11]. The size and concentration of isolated exosomes were determined by Nanosight tracking analysis using ZetaView PMX 110 (Particle Metrix, Inning am Ammersee, Germany), while the expression of AnxA6 and other exosome markers was assessed by Western blotting.

### 2.6. ELISA Assays

The quantification of human CCL2/MCP-1 in TNBC cell lysates, cleared culture supernatants, or isolated exosomes was carried out using DuoSet^®^ Sandwich ELISA Kits for CCL2/MCP-1, according to the manufacturer’s instructions (R&D Systems, Inc., Minneapolis, MN, USA). The reactions were stopped by the addition of 2 N H_2_SO_4_, and the absorbance was measured at 450 nm. The concentration of cytokines was extrapolated from a standard curve.

### 2.7. Co-Immunoprecipitation and Western Blotting

Cells were cultured to 80% confluency in 15 cm dishes, washed twice with ice-cold phosphate-buffered saline (PBS), and treated with or without dithiobis(succinimidyl propionate) (DSP) crosslinker, as previously described [32]. Cells were then harvested by scraping on ice and resuspended in radioimmunoprecipitation assay (RIPA) buffer (50 mM Tris-HCl, pH 7.4, 1% NP-40, 0.1% sodium deoxycholate, 150 mM NaCl, 1 mM EDTA) supplemented with a protease inhibitor cocktail (Sigma-Aldrich, St. Louis, MO, USA) and phosphatase inhibitors (20 mM sodium fluoride, 50 mM β-glycerophosphate, and 0.1 mM sodium ortho-vanadate) and processed as previously described [33]. After centrifugation at >10,000× *g* for 10 min at 4 °C, the protein concentration in the whole cell lysate was determined using the Bradford assay (Bio-Rad Laboratories, Hercules, CA, USA). Immunoprecipitation was performed as previously described [8], and the immune complexes were analyzed by Western blotting using antibodies against the following proteins: AnxA6 (Santa Cruz Biotechnology, Dallas, TX, USA, cat# sc-271859), β-actin (Sigma-Aldrich, St. Louis, MO, USA, cat# A1978), EGFR (Cell Signaling Technology, Danvers, MA, USA, cat# 2646), CD63 (Proteintech, Rosemont, IL, USA Cat# 25682-1-AP), Rab7 clone D7A5 (Cell Signaling Technology, Danvers, MA, USA cat#3777), Flotillin-1 (Santa Cruz Biotechnology, Dallas, TX, USA, cat# sc-25506), SNAP23 (Abcam, Waltham, MA, USA, cat# ab-131242 ER 8538). The blots were revealed by ECL (Perkin Elmer, Waltham, MA, USA), and the intensity of the bands was quantified using ImageJ.

### 2.8. GST Pulldown Assay

Control glutathione S-transferase (GST) and GST-AnxA6 fusion constructs were expressed in *E. coli* BL21 and induced with 1 mM isopropyl-1-thio-β-D-galactopyranoside (IPTG) at 30 °C overnight. The cells were harvested by centrifugation at 4500× *g* for 10 min, and the pellets were resuspended in lysis buffer containing 0.5 mg/mL lysozyme. After incubation for 1 h on ice, the lysates were homogenized by sonication and centrifuged at 12,000× *g* for 45 min. The cleared supernatants containing GST or GST-AnxA6 fusion protein were incubated with glutathione–Sepharose 4B beads (Cytiva, Wilmington, DE, USA) for 2 h at RT, washed 3 times with PBS, and maintained at 4 °C. For GST pulldowns, 2 µg of GST or GST-AnxA6 fusion protein immobilized to glutathione beads were incubated with 500 µg of whole cell extract from MDA-468 cells in lysis buffer with or without 1 mM Ca^2+^. The beads were washed three times in lysis buffer with or without 1 mM Ca^2+^, and the bound proteins were dissociated in SDS sample buffer and analyzed by Western blotting.

### 2.9. Transmission Electron Microscopy

The isolated exosomes in PBS were spotted on Formvar-coated grids (Electron Microscopy Sciences, Morgantown, PA, USA), and, after 15 min, they were fixed with 4% paraformaldehyde for 10 min and washed with PBS. Free aldehyde groups were quenched with 0.15 M glycine in PBS, and the grids were stained with 2% phosphotungstic acid, pH 6.1, and allowed to air-dry. For immunogold staining, the grids were blocked with 5% bovine serum albumin (BSA) in PBS and sequentially incubated with mouse anti-AnxA6 antibody and colloidal gold-conjugated donkey anti-mouse IgG and then stained with 2% phosphotungstic acid. The negatively stained samples were imaged on an FEI Tecnai T12 transmission electron microscope at 100 kV using an AMT CR41 side-mounted CCD camera.

### 2.10. Immunofluorescence Assays

MitoTracker^TM^ Red CMXRos (mitochondria), ER-Tracker^TM^ Red-BODIPY^TM^ TR glibenclamide (endoplasmic reticulum), LysoTracker deep red (lysosomes), and CellMask™ deep red (plasma membrane) organelle tracking dyes were purchased from Thermo Fisher Scientific, Waltham, MA, USA. For live cell imaging with these cell-permeable dyes, cells were plated in 35 mm non-coated glass-bottom dishes, cultured overnight in complete medium, and then transfected with green fluorescent protein (GFP) expressing empty vector control (pEGFP-N1) and GFP-AnxA6 (pEGFP-A6) plasmids using Lipofectamine 3000 transfection reagent (Invitrogen, Waltham, MA, USA). After 48 h, the cells were treated with the indicated organelle tracking dyes according to the manufacturer’s instructions (Thermofisher Scientific, Waltham, MA, USA). Cells were imaged by confocal microscopy using a 60× objective.

### 2.11. Cholesterol Measurements

Cholesterol in the isolated exosomes was determined using equal amounts of protein or equal numbers of EV particles and the Amplex™ Red Cholesterol Assay Kit (Life Technologies, Waltham, MA, USA), according to the manufacturer’s instructions and as previously described [9]. The converted fluorescent resorufin was measured by using a fluorescent microplate reader at 560/590 nm (Ex/Em), and the levels of cholesterol were extrapolated from a cholesterol standard curve.

### 2.12. Proximity Ligation Assay

Cells were plated in 8-well glass-bottom chamber slides and cultured overnight in complete medium. In situ proximity ligation assay (PLA) for AnxA6 and SNAP23 was conducted by using the Duolink^®^ In Situ Red Starter Kit Mouse/Rabbit with mouse anti-AnxA6 (Santa Cruz Biotechnology, Dallas, TX, USA) and rabbit anti-SNAP23 EPR8538 (Abcam Waltham, MA, USA), as described by the manufacturer (Sigma-Aldrich, St. Louis, MO, USA). The reactions were visualized by confocal microscopy using 40× magnification.

### 2.13. Statistical Analysis

Experiments were carried out at least three times and data represented as mean values with standard deviations, unless otherwise indicated. Data were analyzed using Student’s *t*-test or one-way and two-way analysis of variance (ANOVA) in GraphPad Prism 11.0.2 (GraphPad Software Inc., San Diego, CA, USA). For all statistical analyses, *p* < 0.05 was considered statistically significant.

## 3. Results

### 3.1. AnxA6 Influences the Secretion of Pro-Inflammatory Cytokines and Extracellular Vesicles in TNBC Cells

**Figure 1:**
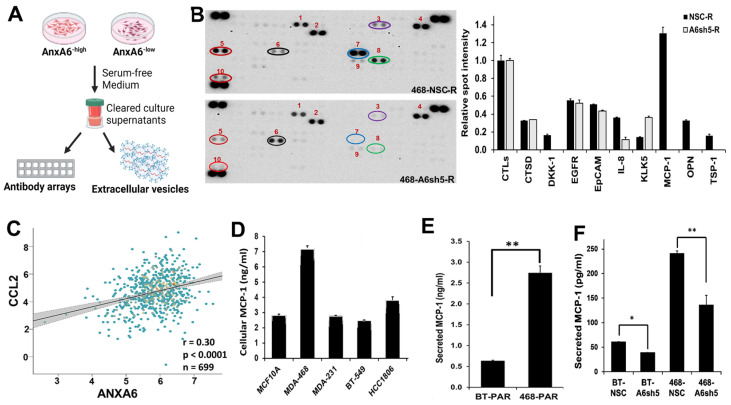
Secretion of pro-inflammatory cytokines is AnxA6-dependent in TNBC cells. (**A**) Schematic showing the experimental design used to harvest cleared 24 h culture supernatants for isolation of extracellular vesicles and proteomic profiling by antibody arrays from AnxA6-high control (NSC) and AnxA6-low or -downregulated (A6sh) MDA-468 cells. (**B**) Detection of secreted proteins in cleared culture supernatants from lapatinib-resistant AnxA6-expressing control (NSC-R) and AnxA6-downregulated (A6sh-R) MDA-468 cells by antibody arrays and densitometric analysis of proteins by ImageJ particle analysis. Bars represent the spot density relative to controls (CTLs). The color-coded circles and numbers refer to the corresponding protein spots in the AnxA6 expressing (468-NSC-R) and AnxA6 depleted (468-A6sh5-R) membranes (**C**) Validation of the co-expression of AnxA6 and MCP-1 in TNBC tumor tissues by Pearson’s pairwise correlation. (**D**–**F**) Relative expression and secretion of MCP-1 in epithelial and mesenchymal-like TNBC cells. Cellular levels (**D**) and secreted (**E**,**F**) MCP-1 were assayed in cell lysates or cleared culture supernatants from the indicated cell lines by ELISA. * denotes *p* < 0.05; ** denotes *p* < 0.01.

In Widatalla et al. [9], we demonstrated that upregulation of AnxA6 and accumulation of cholesterol in late endosomes is a novel mechanism for acquired resistance of TNBC cells to chronic lapatinib (an EGFR/HER2 tyrosine kinase inhibitor) treatment or lapatinib resistance (LAP-R). We also showed, in the same study, that withdrawal of the treatment led to the return of AnxA6 expression to near-basal levels [9]. The current study was conceived in part to investigate the fate of cholesterol in LAP-R TNBC cells. To demonstrate whether the secretion of pro-inflammatory cytokines and extracellular vesicles (EVs) is AnxA6-dependent in TNBC cells, we first profiled secreted proteins in cleared culture supernatants from control AnxA6-expressing (NSC) and AnxA6-downregulated (A6sh) MDA-468 cells by antibody arrays (Figure 1A). As shown in Figure 1B, reduced expression of AnxA6 inhibited the secretion of DKK-1, IL-8, MCP-1, OPN, and TSP-1 but stimulated the secretion of KLK5. Among these factors, the secretion of MCP-1 in MDA-468 cells was strongly inhibited in AnxA6-downregulated cells. Pearson’s pairwise correlation of AnxA6 and CCL2 (MCP-1) from Breast Cancer Gene-Expression Miner (GenExMiner) v3.0 [34] analysis yielded a significant positive correlation (r = 0.30, *p* < 0.0001), suggesting that these proteins are either co-expressed or are relevant in the same regulatory pathway(s) in TNBC subtypes (*n* = 699) (Figure 1C). Although the expression of MCP-1 in TNBC cells is cell type-dependent, prototype epithelial TNBC cell lines such as MDA-468 and HCC1806 express higher levels than model mesenchymal-like TNBC cell lines such as BT-549 and MDA-231 (Figure 1D). Consistent with this observation, the secretion of MCP-1 is higher for parental MDA-468 cells compared to parental BT-549 cells (Figure 1E), and that downregulation of AnxA6 in both BT-549 and MDA-468 TNBC cells strongly reduced the secretion of MCP-1 (Figure 1F). 

**Figure 2:**
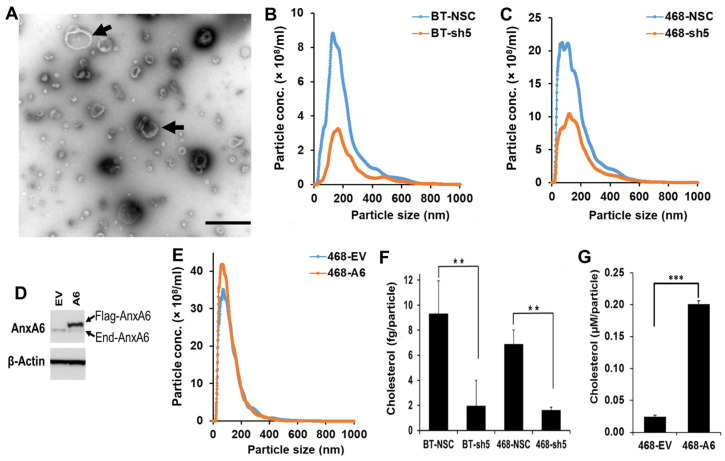
Secretion of small extracellular vesicles is AnxA6-dependent in TNBC cells. Extracellular vesicles (EVs) were purified from culture supernatants from control (NSC) and AnxA6-downregulated (sh5) BT-549 or MDA-468 TNBC cells and from empty vector control and AnxA6-upregulated MDA-468 cells. (**A**) Verification of isolated EVs by transmission electron microscopy. Scale bar: 100 nm. Arrows indicate the cup-shapped EVs of varying sizes. (**B**,**C**,**E**) Nanoparticle tracking analysis of isolated EVs. (**D**) Western blot showing overexpression of AnxA6 in MDA-MB-468 cells. β-actin was used as the loading control. (**F**,**G**) Determination of cholesterol in EVs isolated from control and AnxA6-downregulated TNBC cells (**F**) and empty vector control and AnxA6-overexpressing MDA-468 cells (**G**). Bars represent EV-associated cholesterol/particle. End-AnxA6: endogenously expressed AnxA6; Flag-AnxA6: recombinant Flag-tagged AnxA6. ** denotes *p* < 0.01; *** denotes *p* < 0.001.

To determine if AnxA6 expression status also influences the secretion of small EVs (exosomes), we isolated EVs from cleared culture supernatants of control AnxA6-expressing and AnxA6-downregulated BT-549 and MDA-468 TNBC cells by differential velocity centrifugation [27]. Figure 2A (arrows) shows the transmission electron micrographs of isolated EVs. Analysis of the isolated EVs byusing the ZetaView^®^ Nanoparticle Tracking Analyzer revealed that downregulation of AnxA6 strongly reduced the secretion of EVs in both BT-549 and MDA-468 cells (Figure 2B,C). Upregulation of AnxA6 in the AnxA6-low MDA-468 cells (Figure 2D), on the contrary, led to increased secretion of EVs (Figure 2E). Assay of cholesterol in the isolated EVs also revealed that downregulation of AnxA6 in these cell lines is associated with decreased EV-associated cholesterol (Figure 2F), while upregulation of AnxA6 in MDA-468 cells led to increased EVs-associated cholesterol (Figure 2F). Together, this suggests that AnxA6 expression is associated with increased secretion of cholesterol enriched EVs in TNBC cells.

**Figure 3:**
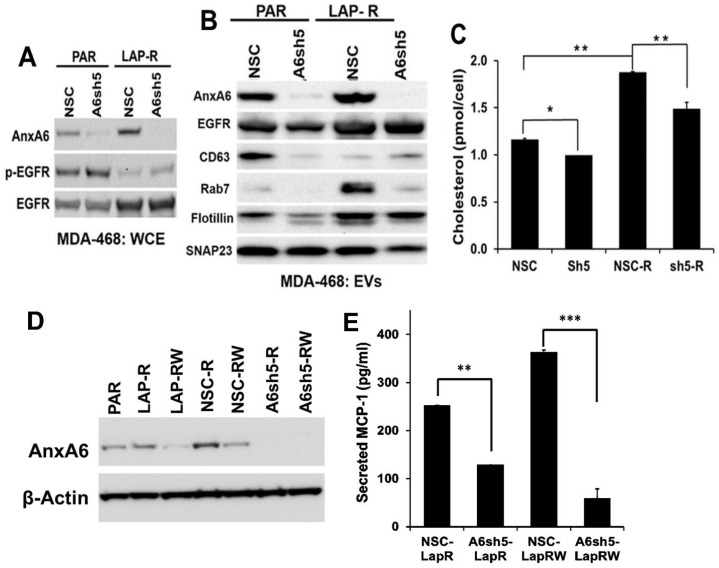
Lapatinib-induced expression of AnxA6 promotes the secretion of AnxA6 and cholesterol-enriched EVs. (**A**) Western blot showing inhibition of EGFR activation in MDA-468 cells following chronic treatment with lapatinib. (**B**) Enrichment of AnxA6 and Rab7A in EVs from lapatinib-resistant MDA-468 cells. (**C**) AnxA6-dependent enrichment of cholesterol in lapatinib-resistant MDA-468 cells. Bars represent cholesterol levels from two independent determinations. (**D**) Western blot analysis of AnxA6 expression in parental, lapatinib resistant and lapatinib withdrawn control AnxA6 expressing and AnxA6-depleted MDA-468 cells. (**E**) Lapatinib withdrawal stimulated the secretion of MCP-1 in AnxA6-expressing NSC cells (c.f. NSC-LapR and NSC-LapRW) and decreased secretion in AnxA6-downregulated A6sh5-LapR and A6sh5-LapRW. * denotes *p* < 0.05; ** denotes *p* < 0.01; *** denotes *p* < 0.001.

We have previously reported that chronic lapatinib treatment of AnxA6-low MDA-468 TNBC cells led to AnxA6 upregulation and accumulation of cholesterol in late endosomes [9]. To determine if chronic lapatinib-induced expression of AnxA6 is associated with increased secretion of EVs and proinflammatory cytokines, we first confirmed that chronic treatment of MDA-468 (LAP-R) cells with lapatinib strongly inhibited EGFR activation and induced AnxA6-expression (Figure 3A). Analysis of the isolated EVs by Western blotting revealed that AnxA6 and the small Ras-related GTPase Rab7 were enriched in EVs isolated from lapatinib-treated cells (Figure 3B). This analysis also showed that flotillin, an exosome marker, was enriched in lapatinib-resistant cells while CD63 was strongly downregulated in AnxA6-depleted and lapatinib-resistant cells (Figure 3B). We also showed that EV-associated cholesterol levels were higher in control AnxA6-expressing cells compared to AnxA6-downregulated cells, and, more importantly, the cholesterol levels in lapatinib-treated cells (with increased AnxA6 expression) were significantly higher in LAP-R cells than in the untreated control cells (Figure 3C). We next showed that the secretion of MCP-1 was also AnxA6-dependent following lapatinib treatment (LAP-R) and that the withdrawal of lapatinib from lapatinib-resistant cells, denoted LAP-RW (Figure 3D), that led to near-basal levels of AnxA6 [9], was accompanied by significantly higher secretion of MCP-1 in the AnxA6 expressing NSC-LapRW cells compared to the corresponding AnxA6 depleted A6sh5-LapRW cells (Figure 3E). Together, these observations confirm data in Figure 1 and Figure 2 showing that AnxA6 downregulation inhibited cholesterol enrichment in EVs and that lapatinib-induced expression of AnxA6 promotes MCP-1 secretion.

### 3.2. AnxA6 Influences the Secretion of Pro-Inflammatory Cytokines and Exosomes via Interaction with SNAP23

**Figure 4:**
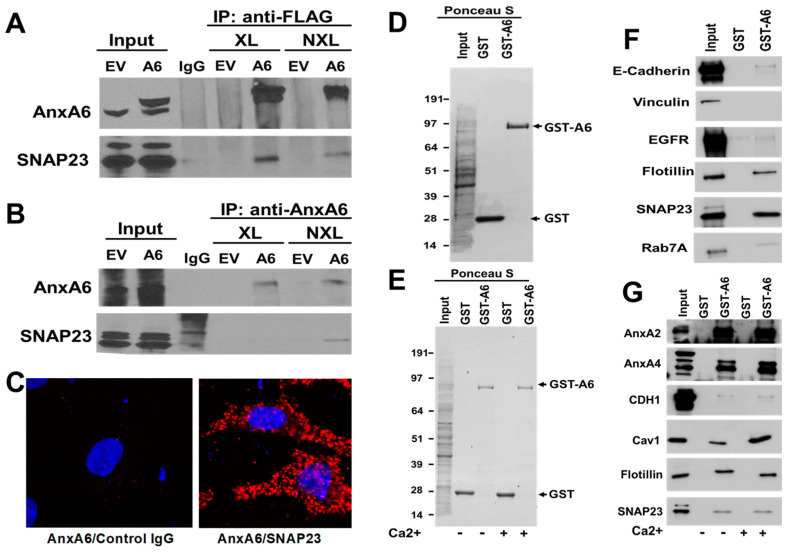
AnxA6 interacts with the SNARE protein SNAP23 in TNBC cells. (**A**,**B**) Representative blots from co-immunoprecipitation of AnxA6 and SNAP23 from cell lysates prepared from empty vector (EV) and Flag-AnxA6 (A6)-transfected MDA-468 cells with (XL) or without (NXL) the crosslinking agent DSP. Immunoprecipitation was carried out using control IgG and either anti-Flag M2 antibody (**A**) or anti-AnxA6 antibody (**B**). Detection of AnxA6 and SNAP23 in the immune complexes was carried out by Western blotting. (**C**) Validation of AnxA6 and SNAP23 interaction by proximity ligation assay (PLA) using mouse anti-AnxA6 and rabbit anti-SNAP23. Normal rabbit IgG and mouse ati-AnxA6 were used as controls. (**D**–**G**) Validation of the interaction of AnxA6 with SNAP23 by GST pulldown assay. Equal amounts of purified GST and GST-AnxA6 were incubated with equal amounts of cell lysates from MDA-468 cells (**D**,**F**) and in GST pulldown buffer with or without 1 mM Ca^2+^ (**E**,**G**). Ponceau S-stained membranes show the expression of GST and GST-A6 (**D**,**E**). Blots were probed with antibodies against the indicated proteins by Western blotting (**F**,**G**). NXL: non-crosslinked, XL: crosslinked with DSP.

Co-immunoprecipitation and Western blotting using anti-Flag-M2 antibody (Sigma-Aldrich, St. Louis, MO, USA) and whole cell lysates from MDA-468 cells expressing Flag-tagged AnxA6 revealed that AnxA6 interacted with members of the SNARE (soluble N-ethylmaleimide-sensitive factor (NSF) attachment protein receptor) membrane fusion complex [35], including SNAP23. AnxA6 and SNAP23 interacted when immunoprecipitated with either anti-Flag-M2 (Figure 4A) or anti-AnxA6 (Figure 4B) antibodies. The interaction of AnxA6 and SNAP23 was stable as this was detected without crosslinking with DSP. To validate this, we performed a proximity ligation assay using mouse anti-AnxA6 antibody (Santa Cruz Biotechnology, Dallas, TX, USA) and rabbit anti-SNAP23 antibody (Abcam, Waltham, MA, USA) and confirmed that AnxA6 strongly interacted with SNAP23 (Figure 4C). Furthermore, GST pulldown assays also confirmed that AnxA6 interacted with SNAP23 as well as other potential AnxA6-interacting proteins, including flotillin, E-cadherin, EGFR, and Rab7 (Figure 4D). The interaction of these proteins with AnxA6 appeared to be Ca^2+^-independent (Figure 4E). Together, these data suggest that AnxA6 interacts with at least one component of the SNARE complex, with implications in the secretion of pro-inflammatory cytokines and EVs in TNBC cells.

**Figure 5:**
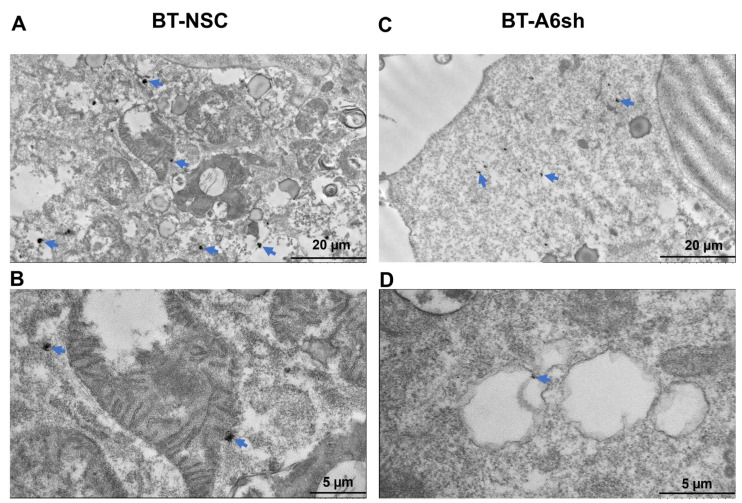
Subcellular localization of AnxA6 in TNBC cells. Cells were processed and analyzed by immunogold transmission electron microscopy. Images are representative transmission electron micrographs at the same magnification from AnxA6-expressing BT-NSC (**A**,**B**) and AnxA6-downregulated BT-A6sh (**C**,**D**) BT-549 cells. Arrows indicate the subcellular location of AnxA6 within each cell.

We next used immunogold transmission electron microscopy to determine the localization of AnxA6 in TNBC cells. Consistent with our previous report [12], the subcellular imaging revealed that, in AnxA6-expressing TNBC cells, AnxA6 is aggregated on several membrane-bound organelles (Figure 5A,B, blue arrowheads). In AnxA6-downregulated cells, the residual AnxA6 appeared as less intense puncta (Figure 5C,D).

**Figure 6:**
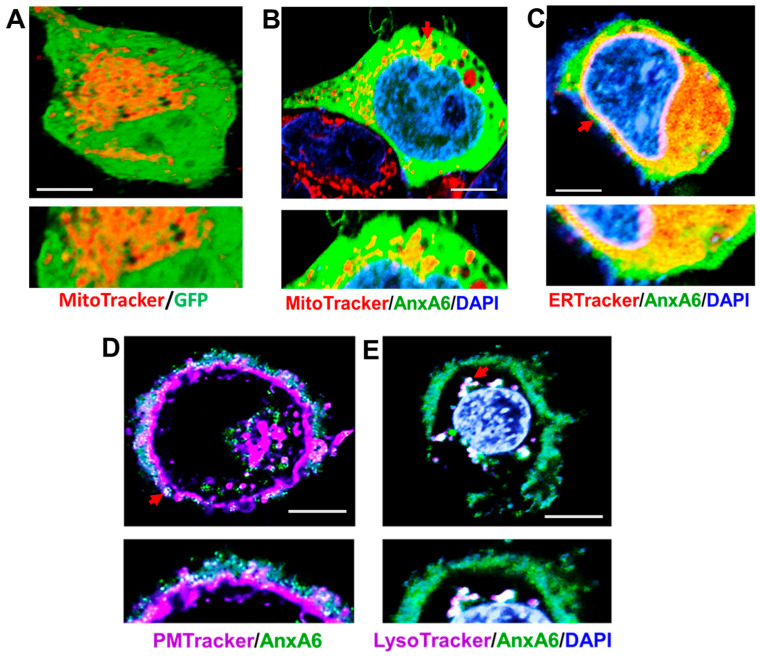
Localization of AnxA6 on membrane-bound cellular structures. Control GFP (**A**) and GFP-AnxA6 (**B**–**E**) transfected BT-549 cells were incubated with the indicated organelle tracking dyes as described in Section 2. Shown are representative merged images at 60× magnification to visualize AnxA6 in mitochondria (**A**,**B**), the endoplasmic reticulum (**C**), the plasma membrane (**D**), and lysosomes (**E**). Insets represent enhanced sections of the images to emphasize co-localization (red arrowheads). Scale bars: 50 µm.

To confirm that AnxA6 localizes to several membrane-bound organelles, we used confocal microscopy to co-localize organelle tracking dyes in GFP-AnxA6-transfected cells. Relative to the GFP control (Figure 6A), this analysis confirmed the localization of AnxA6 in mitochondria (Figure 6B) and the endoplasmic reticulum (Figure 6C), depicted by the yellow regions in the merged images, as well as in the plasma membrane (Figure 6D) and lysosomes (Figure 6E), depicted by the white merged images. These data support the relevance of AnxA6 in various membrane-associated processes, including the trafficking of intracellular vesicles from the endosomal compartment and membrane dynamics of organelles such as mitochondria and lysosomes, as well as exocytosis/endocytosis at the plasma membrane.

### 3.3. Extracellular AnxA6 Is Required for the Survival of Basal-like Epithelial TNBC Cells

**Figure 7:**
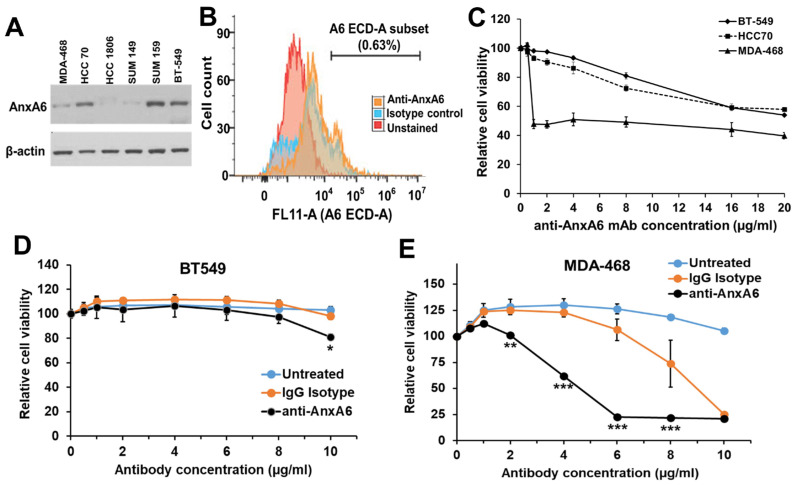
Extracellular AnxA6 is critical for the survival of basal-like epithelial TNBC cells. (**A**) Expressioin of AnxA6 in a panel of TNBC cells by Western blotting. (**B**) Detection of extracellular AnxA6 by flow cytometry. A6 ECD-A subset: extracelluar AnxA6 positive cells. (**C**–**E**) Differential susceptibility of TNBC cells expressing high (**C**,**D**) and relatively low (**C**,**E**) levels of AnxA6 following treatment with AnxA6-neutralizing antibodies. * denotes *p* < 0.05; ** denotes *p* < 0.01; *** denotes *p* < 0.001.

Although AnxA6 is a predominantly intracellular protein, it is also detected in the extracellular space [12]. To determine if extracellular AnxA6 affects the growth of TNBC cells, we assessed the effects of a mouse monoclonal anti-Annexin VI (Antibody IgG_2b_ κ, clone G-10; Santa Cruz Biotechnology, Dallas TX, USA, cat#SC-166807) as neutralizing AnxA6 antibodies. Western blotting revealed that BT-549 and HCC70 expressed relatively high levels of AnxA6, while MDA-468 cells express relatively low levels of the protein (Figure 7A). Consistent with previous studies [12], we next showed that the antibody recognized AnxA6 on the surfaces of BT-549 cells (Figure 7B) and that, although anti-AnxA6 antibodies dose-dependently reduced the viability of TNBC cells, HCC70 and BT-549 cells that expressed relatively high levels of AnxA6 were more resistant than MDA-468 cells that expressed relatively low levels of AnxA6 (Figure 7C). We next compared the effects of the neutralizing antibodies on the isotype control (IgG2b κ) and showed that the viability of BT-549 cells was only slightly decreased by 10 µg/mL (Figure 7D), while that of MDA-468 was significantly reduced by 2 µg/mL (range 2–6 µg/mL (Figure 7E)). Together, this suggests that extracellular AnxA6 may be required for anchorage for the survival of proliferative AnxA6-low TNBC cells. However, additional studies are warranted to ensure that the effects are due to reduced availability of extracellular AnxA6 and not antibody-mediated cytotoxicity.

## 4. Discussion

Thus far, substantial evidence supports the role of annexins, including AnxA6, in the formation of membrane contact sites (MCSs) and intraluminal vesicles (ILVs) and the transport of cholesterol from late endosomes [26]. Several studies have also reported the potential for AnxA6-enriched exosomes/EVs to influence the progression, drug resistance, and metastasis of breast, pancreatic, and other cancers [4,14,15,16]. Our study therefore demonstrates that the secretion of EVs and pro-inflammatory cytokines such as MCP-1, as well as cholesterol enrichment in EVs, is AnxA6-dependent. The downregulation of AnxA6 inhibited, while the overexpression or chronic lapatinib-induced expression of AnxA6 promoted, the secretion of MCP-1 and enrichment of cholesterol in EVs. This study also shows that AnxA6 influences the secretion of these factors by its Ca^2+^-dependent translocation to the plasma membrane and several membrane-bound organelles and that its interaction with SNAP23, a critical component of the SNARE membrane fusion machinery [36], supports, at least in part, its involvement in the secretion of these factors. Given that AnxA6 is also detected extracellularly and is associated with EVs [11,12,27], the decrease in cell survival following treatment of TNBC cells with neutralizing anti-AnxA6 antibodies supports the notion that extracellular and/or EV-associated AnxA6 could be targeted to attenuate basal-like breast cancer progression, metastasis, and drug resistance. Further studies are warranted to unequivocally demonstrate the potential to block AnxA6 in various TNBC cellular subtypes.

Although AnxA6-low TNBC cells are typically highly proliferative, the relationship between rapid cell proliferation and low AnxA6 cellular levels remains poorly understood. As a scaffolding protein, AnxA6 may promote tumor cell proliferation via multiple mechanisms. Previous studies have implicated increased expression and activity of Ras-specific guanine nucleotide releasing factor 2 (RasGRF2) in the proliferative phenotype [8]. This study suggests that the requirement for extracellular AnxA6 in the survival of basal-like epithelial TNBC cells is consistent with its role in the secretion of cytokines from secretory granules and EVs from MVBs. Although this remains to be thoroughly investigated, AnxA6 may also be critical for the uptake of EVs by recipient cells.

The requirement for Ca^2+^ in AnxA6-mediated exocytosis/secretion of EVs has thus far been shown to depend on the cell type and/or pool of secretory molecules. In HCT116 colon cancer cells, AnxA6 is recruited to MVBs in the presence of Ca^2+^ and AnxA6 is required for Ca^2+^-dependent exosome secretion both in intact and in permeabilized cells [28]. This study specifically demonstrated the requirement for AnxA6 in the plasma membrane damage repair-induced secretion of exosomes [28]. On the contrary, other reports have suggested that membrane-proximal lysosomes rather than multivesicular bodies (MVBs) are responsible for Ca^2+^-dependent exocytosis in non-stimulatory cells [37,38]. Despite these contradictions, rapid release of EVs has been linked to elevated levels of cytosolic Ca^2+^ in T cells and leukemia cells [39,40]. Our immunogold transmission electron micrographs support the notion that, in AnxA6-expressing cells, extracellular Ca^2+^ mediated increase in cytosolic Ca^2+^ triggered the translocation of AnxA6 to the plasma membrane and several membrane bound compartments. This effect was markedly reduced following AnxA6 downregulation and associated with decreased secretion of both EVs and proinflammatory cytokines.

Earlier studies reported two modes of Ca^2+^-dependent association of AnxA6 with cell membranes and suggested the involvement of AnxA6 in secretion/exocytosis. At low Ca^2+^ concentrations (up to 150 µM), AnxA6 is thought to bind to the same membrane via its two coplanar annexin core domains, but, at high Ca^2+^ concentrations (≥2 mM), AnxA6 binds to two adjacent phospholipid membranes with positive cooperativity [41,42]. However, the argument for the negative regulation of secretion by AnxA6 is based on the observation that AnxA6 more effectively inhibited synexin (AnxA7)-induced granule aggregation in chromaffin cells. In another study in PC12 cells, overexpression of AnxA6 isoforms almost completely inhibited dopamine secretion, while AnxA6 knockdown in these cells was accompanied by a 20% enhancement [30]. In Chinese hamster ovary cells, the expression of AnxA6 led to sequestration of cholesterol in the late endosomal compartment and reduced cholesterol in the plasma membrane, which negatively affected secretion [3]. These reports together emphasize the notion that the subcellular distribution of cholesterol is strongly influenced by the expression levels and subcellular localization of AnxA6 [43,44].

Thus far, the involvement of AnxA6 in the secretion of cytokines is poorly understood and limited to IL-2 secretion studies in Jurkat T cells. In these cells, the intracellular localization of AnxA6 switched from the cytosol to vesicular structures near the plasma membrane following an increase in intracellular Ca^2+^ or decrease in acidity, suggesting that AnxA6 promotes the Ca^2+^- and proton-dependent secretion of cytokines [45]. This is supported by the observation that T cells isolated from AnxA6-deficient mice exhibited reduced membrane order, low cholesterol levels, and reduced interleukin-2 (IL-2) signaling compared with T cells derived from wild-type mice [46]. Similar observations have been reported following cholesterol depletion with methyl-β-cyclodextrin and statins, which led to a decreased chemotactic response of monocytes to MCP-1 [47], and cholesterol-rich oxidized low-density lipoprotein (OxLDL)-bound MCP-1 retained its ability to recruit monocytes [48]. While this supports the role of cholesterol in MCP-1 secretion and chemotactic functions, our finding that the secretion of MCP-1 as well as AnxA6 and cholesterol-enriched EVs is AnxA6-dependent further suggests that AnxA6 and cholesterol together influence the secretion and activity of cytokines.

The mechanism by which AnxA6 influences the secretion of cytokines and/or EVs remains poorly understood. Previous studies have reported the mislocalization of components of the SNARE (soluble N-ethylmaleimide-sensitive fusion protein attachment protein receptors) complex, including SNAP23, STX4, and VAMP8, with accumulation of cholesterol in late endosomes [49]. Other studies have suggested the regulation of the SNARE complex by several factors, denoted as tethering factors, that facilitate the fusion of vesicular and plasma membranes [50]. The interaction of AnxA6 with SNAP23, as demonstrated in this study, links AnxA6 to the SNARE complex—the major membrane fusion machinery that is critical for secretion/exocytosis [36]. A role for AnxA6 in secretion is supported by this study and a recent report showing that secretion of exosomes is stimulated in damaged cells and that reduced expression of AnxA6 led to accumulation of MVBs in the cell periphery [28]. Although the AnxA6-SNAP23 interaction is validated and verified by co-immunoprecipitation, GST-pull down and proximity ligation assays, this finding is by no means an indication of how AnxA6 influences the SNARE complex or the secretion of cytokines and exosomes. Given the potential role that Ca^2+^ plays in the fusion of secretory vesicles or MVBs with the plasma membrane for exocytosis [39] and the Ca^2+^-dependent translocation of AnxA6 to cellular membranes [12], the interaction of AnxA6 with SNAP23 may be facilitated by Ca2+-dependent translocation of AnxA6 to membrane fusion sites containing the SNARE complex on the plasma membrane. However, further studies are necessary to unequivocally demonstrate how AnxA6 influences SNAP23 and/or the SNARE complex assembly and functions in the release of cytokines and/or EVs.

Previous studies on AnxA6 and TNBC biology have led to the concept that cells expressing relatively high levels of AnxA6 grow poorly and are inherently drug-resistant, morphologically mesenchymal-like, and highly migratory, with a lipogenic metabolic phenotype. Meanwhile, AnxA6-low cells are highly proliferative epithelial cells that migrate poorly, are drug-sensitive, and exhibit a lipolytic metabolic phenotype [6,8,9,10,12]. This somewhat paradoxical involvement of AnxA6 in TNBC progression is intriguing. As a predominantly intracellular scaffolding protein with no enzymatic activity, AnxA6 has been shown to influence tumor growth, metastasis, and drug resistance [1], but its therapeutic targeting has not been sufficiently explored. The decrease in the survival of AnxA6-low TNBC cells but not tumor cells expressing relatively high levels of AnxA6 by monoclonal anti-AnxA6 neutralizing antibodies suggests the requirement for extracellular AnxA6 in the growth of these tumor cells. This is consistent with previous studies in which anti-AnxA2 antibodies were demonstrated to significantly decrease the invasion of chick embryo chorioallantoic membranes, as well as tumor growth and metastasis in breast, ovarian, and pancreatic cancers [51,52,53]. While some of these effects are mediated via annexins as membrane repair proteins [28,54], our data suggest that reducing the levels of extracellular AnxA6 with anti-AnxA6 neutralizing antibodies may be a viable therapeutic strategy to target this scaffolding protein, especially in the AnxA6-low, highly proliferative basal-like breast cancer.

Reduced expression levels of AnxA6 have been shown to be accompanied by a corresponding decrease in the expression of several membrane proteins, including receptor tyrosine kinases, e.g., EGFR, the immune checkpoint marker PD-L1, the stem cell markers EpCAM and CD44, and the hypoxia-inducible pH sensor carbonic anhydrase 9 [10,55]. Reduced expression of AnxA6 has also been shown to affect the function of membrane microdomains such as focal adhesions [12], which are critical for anchorage-dependent cell growth and cell motility [56,57]. Given that downregulation of AnxA6 sensitizes cells to tyrosine kinase inhibitors, androgen receptor (AR) antagonists, and chemotherapy [6,9,10,31], and that chronic treatment of AnxA6-low cells with these agents, including hypoxia [7], reactivates the expression of AnxA6 as a mechanism for resistance to treatment [9], it is possible that blocking extracellular AnxA6 may render tumor cells more vulnerable to treatment with these agents. However, further studies are needed (a) to validate the finding with or without agents targeting these AnxA6-modulated membrane proteins; (b) to ascertain if the reduced adhesion and motility following the RNAi-mediated knockdown of AnxA6 is also due to extracellular AnxA6; and (c) to decode the extracellular AnxA6-induced cell death mechanism(s) for more viable treatment strategies for TNBC.

## 5. Conclusions

In summary, our data reveal that extracellular AnxA6 may be important for the survival of highly proliferative basal-like TNBC cells. Our data also suggest that AnxA6 Ca^2+^-dependently translocates to fusion competent membrane microdomains, at which its interaction with SNAP23 presumably facilitates the secretion of cytokines/exosomes. Overall, these data suggest that AnxA6 promotes TNBC progression at least in part by facilitating the secretion of pro-inflammatory cytokines and cholesterol-enriched exosomes. Further studies are necessary to determine if AnxA6 is necessary for regulated or constitutive secretion of exosomes and/or pro-inflammatory cytokines and to determine the relevance of the interaction of AnxA6 with SNAP23 in Ca^2+^-stimulated exocytosis.

## Data Availability

All data are available in the main text or from the corresponding author. Graphical raw data and statistical analyses are included in the source data files.
